# The wild tomato species *Solanum chilense* shows variation in pathogen resistance between geographically distinct populations

**DOI:** 10.7717/peerj.2910

**Published:** 2017-01-18

**Authors:** Remco Stam, Daniela Scheikl, Aurélien Tellier

**Affiliations:** Section of Population Genetics, Technical University of Munich, Freising, Germany

**Keywords:** Wild tomato, Phytopathology, Fusarium, Phytophthora, Alternaria, General liniarised mixed models

## Abstract

Wild tomatoes are a valuable source of disease resistance germplasm for tomato (*Solanum lycopersicum*) breeders. Many species are known to possess a certain degree of resistance against certain pathogens; however, evolution of resistance traits is yet poorly understood. For some species, like *Solanum chilense*, both differences in habitat and within species genetic diversity are very large. Here we aim to investigate the occurrence of spatially heterogeneous coevolutionary pressures between populations of *S. chilense*. We investigate the phenotypic differences in disease resistance within *S. chilense* against three common tomato pathogens (*Alternaria solani*, *Phytophthora infestans* and a *Fusarium sp*.) and confirm high degrees of variability in resistance properties between selected populations. Using generalised linear mixed models, we show that disease resistance does not follow the known demographic patterns of the species. Models with up to five available climatic and geographic variables are required to best describe resistance differences, confirming the complexity of factors involved in local resistance variation. We confirm that within *S. chilense*, resistance properties against various pathogens show a mosaic pattern and do not follow environmental patterns, indicating the strength of local pathogen pressures. Our study can form the basis for further investigations of the genetic traits involved.

## Introduction

In nature, plants are exposed to a wide range of pathogens and pests. While in most cases the plants appear non-specifically resistant against these threats, drastic or recurrent epidemics do occur (Thrall, Burdon & Bock, 2001; Soubeyrand et al., 2009) and variability in specific resistance to pathogens is observed (Thrall, Burdon & Young, 2001; Salvaudon, Giraud & Shykoff, 2008). Understanding how reciprocal co-adaptation of hosts and pathogens maintains such diversity has been a key question in theoretical and empirical evolutionary biology. Theoretically, negative direct frequency-dependent selection (ndFDS) is shown to be a necessary condition to maintain long-term stable diversity for resistance in plants and infectivity in pathogens (Tellier & Brown, 2007). Seed banking, perenniality or polycyclic disease can generate ndFDS, while costs of resistance and infectivity (virulence) are necessary but not sufficient for stable long term polymorphism to occur (Tellier & Brown, 2009; Brown & Tellier, 2011). Another factor often suggested to maintain diversity is the spatial structure of host and pathogen populations. Spatial structure and migration of hosts and pathogens as well as population sizes and genetic drift generate patterns of local adaptation over space and time (Thrall & Burdon, 2002; Gandon & Michalakis, 2002). However, a spatial structure with homogeneous environment does not generate ndFDS (Thrall, Burdon & Bever, 2002; Tellier & Brown, 2011). Stable long term polymorphism is favoured by spatially heterogeneous environments across which the prevalence and severity of disease or the costs of resistance and infectivity may differ (Gavrilets & Michalakis, 2008; Moreno-Gamez, Stephan & Tellier, 2013).

From an ecological perspective, and based on the classic disease triangle from plant pathology (Agrios, 2005) the outcome of species interactions are mediated by the abiotic and biotic environment. The influence of the environment therefore generates spatial and temporal variation in evolutionary and coevolutionary dynamics (Thompson, 2005), and increasing evidence for geographical variation in coevolutionary dynamics and patterns of local adaptation are found in microcosm experiments (Forde, Thompson & Bohannan, 2004; Vogwill et al., 2009; Lopez-Pascua, Brockhurst & Buckling, 2010).

Nevertheless, few field systems exist to study and document the coevolution of plants and their pathogens occurring at short time scales and across several populations. One example is the wild flax—flax rust pathosystem, where local adaptations have been observed and the most resistant varieties of flax generally harboured more virulent strains of rust (Thrall, Burdon & Bever, 2002; Thrall & Burdon, 2003). Similarly, the local adaptation of powdery mildew *Podosphaera plantaginis* to * Plantago lanceolata* populations spread over different islands off the coast of Sweden showed virulent strains to co-occur with more resistant plants (Laine, 2005; Soubeyrand et al., 2009). In the latter plant-pathogen system, several theoretically proposed mechanisms to generate ndFDS have been shown to originate from the environmental heterogeneity across populations: (1) GxGxE interactions (host genotype x pathogen genotype x environment, (for example Laine, 2006), (2) heterogeneity in disease incidence and prevalence determining epidemiological pressures (Soubeyrand et al., 2009) * and* co-infection (Susi et al., 2015) and (3) different strength of connectivity between populations accelerating or decelerating the speed of coevolution across the landscape (Jousimo et al., 2014). Thus, these factors are expected to promote and facilitate long term polymorphism at resistance and infectivity loci without unrealistic costs of these alleles. Here we aim to investigate the variation of disease resistance properties amongst populations of *S. chilense,* a wild tomato species, against several pathogens.

Wild *Solanum* species are in general particularly good model species to study between and within species variation, because they occur in diverse geographic and climatic habitats and have a very well studied demography and known evolutionary history (Städler, Roselius & Stephan, 2005; Städler, Arunyawat & Stephan, 2008; Tellier et al., 2011). Additionally, several studies exist suggesting that bacterial resistance-associated genes are under selective pressure (Rose et al., 2005; Rose et al., 2011; Rose, Michelmore & Langley, 2007). *S. chilense* is native in South America, ranging from southern Peru to central Chile, and colonised a broad range of habitats. *S. chilense* populations are found from coastal regions, even in slightly alkaline environments, all the way to high altitude (>3,000 m) mountain regions. It also occurs in extreme dry habitats on the border of the Atacama dessert, as well as near rivers and creeks (Peralta, Spooner & Knapp, 2008).

*S. chilense* most likely originated with its sister species *S. peruvianum,* in south Peru and then migrated south (Städler, Arunyawat & Stephan, 2008). A study of the species’ demography found four genetically distinct groups; one in the north of the range, one in the central region and two in the south (one on the coast and one at high altitudes). Interestingly, the two southern groups are, even though geographically close to each other, more related to the central group than to each other, possibly due to the separating effect of the extremely arid Atacama desert (Böndel et al., 2015). In addition, *S. chilense* shows clear climatic adaptations. Populations from drier regions are responding faster to drought (Fischer et al., 2013) and individual populations found at high altitudes (>3,000 m) show higher freezing tolerance (Nosenko et al., 2016) *S. chilense* has also been the source of resistance loci against the fungus *Verticilium dahliae* (Tabaeizadeh et al., 1999) and against various viruses (Griffiths & Scott, 2001; Ji et al., 2007; Verlaan et al., 2013). Seeing that *S. chilense* occurs in such a wide range of habitats and that the species shows specific signs of local climatic adaptations, we wondered whether we could find variation for pathogen resistance as well.

Since no detailed data exist about the co-occurrence of wild pathogens and *S. chilense*, we chose to test *S. chilense* disease resistance properties with three widely studied and economically relevant pathogens, *Alternaria solani*, *Phytophthora infestans* and a *Fusarium* sp.

*A. solani* causes early blight and is amongst the most destructive diseases of tomato in tropical and subtropical regions, leading to yield losses of up to 80% in certain regions. *A. solani* has been found in central Peru and is known to cause disease not only on potato—its main host—but also on many other nightshades, including tomato (Song et al., 2011; Kumar et al., 2013). In addition, previous work has shown that *A. solani* resistance can be studied using detached leaf assays (Chaerani & Voorrips, 2006; Chaerani et al., 2007).

*Fusarium* spp. are pathogens that cause very severe disease symptoms on a very wide range of host plants that span almost the entire globe (Agrios, 2005). Two *Fusarium* spp are in the top ten most important fungi in plant pathology (Dean et al., 2012). The *F. oxysporium* species complex comprises over 100 formae specialis that all infect specific hosts, including tomato (Michielse & Rep, 2009). The *F. oxysporium* species complex is widely used to study molecular and genetic mechanisms involved in plant pathogen interactions (Houterman, Cornelissen & Rep, 2008; Ma, Cornelissen & Takken, 2013) and even though it is generally reported to be a vascular pathogen, it has regularly been successfully deployed in detached leaf infection assays (e.g., Kavroulakis et al., 2007).

*Phytophthora infestans* is an oomycete that causes late blight on potato and tomato. In potato alone the damage amounts up to $1 bn annually (Haverkort et al., 2009). Due to its economic value and the vast amount of molecular and genetic research performed on it, it is considered the most important oomycete plant pathogen (Kamoun et al., 2015). Like the other two pathogens used in this study, *P. infestans* is known to occur in the geographic range of various wild *Solanum* species (Perez et al., 2001). Specifically, the strain EC1 that we used here has its origin in Ecuador and is particularly relevant for agriculture as it is a rather aggressive strain that is capable of overcoming certain novel genetic resistances (Foster et al., 2009; Nowicki et al., 2011).

**Figure 1:**
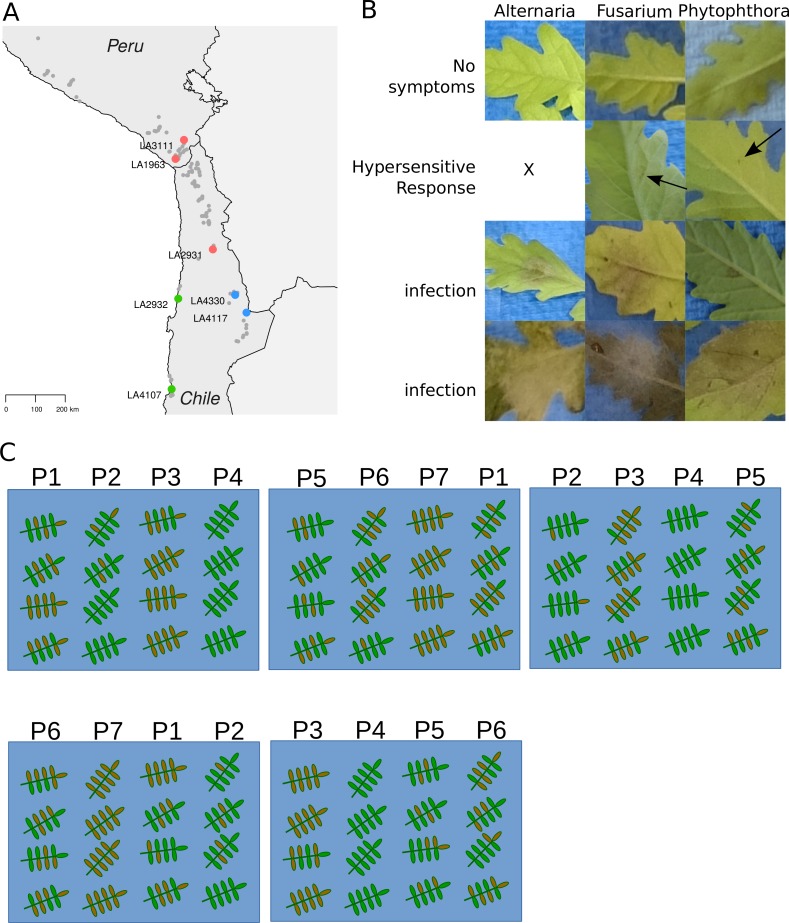
*S. chilense* populations and phenotypic observations. (A) A map showing the populations used in this study, belonging to the central (red), southern mountainous (blue) or southern coastal (green) region. The geographic range of the whole species is depicted in the background (grey dots). (B) The phenotypic observations after infection range from no visible symptoms (No Symptoms row) and small black necrotic lesions resembling the Hypersensitive Response (HR), both scored as ‘not infected,’ to intermediate and strong infection (third and fourth rows: infection and infection, respectively), both scored as ‘infected.’ Columns indicate infection with *Alternaria, Fusarium and Phytophthora*. We could not observe HR in the *Alternaria* infections. (C) Experimental set-up. Detached leaves were randomised for each population (P#) and spread over various boxes. Each leaflet was drop inoculated and scored. Here only the first five boxes are drawn.

Here we test the resistance of different *S. chilense* populations. We selected seven populations that represent three previously described genotype groups from Peru and Chile (Böndel et al., 2015). Two populations originate from the central range (LA1958, LA3111), two from the coastal regions (LA2932, LA4107) and two from the southern mountainous region (LA4117, LA4330). A seventh population is geographically in the middle between the southern mountain and the central group (LA2931). Böndel et al. group it with the central populations, but assign properties of both groups to it. Figure 1A shows the species distribution and highlights the selected populations. These groups correspond to very distinctive habitats and can thus be used to investigate whether we see differences in infection rate throughout the range of the species. We also test whether these differences show a linear pattern when tested against geographical and climatic variables (e.g., corelation between latitute or precipitation and resistance) or whether a multitude of factors leads to specific geographical variations in resistance to each of the three pathogens. Our study will help inform on the occurrence of spatially heterogeneous coevolutionary pressures between the populations of *Solanum chilense*, a solanaceous wild species, and its pathogens.

## Methods

### Plant growth

Seed batches were obtained from the tomato genomics resource centre (TGRC, Davis, USA). We grew seven different *Solanum chilense* populations (accession numbers LA1963, LA2931, LA2932, LA3111, LA4107, LA4117 and LA4330) consisting of 10 different plants each and one *Solanum pennellii* (LA0716) population in our glasshouse from randomly chosen seeds. The plants were grown with 16 h light and a minimum temperature of 18 °C. Mature plants were cut back at a biweekly interval to assure young leaves of similar age were available at all times for all populations.

### Pathogen propagation and spore production

#### Alternaria solani

*A. solani* strains B055* and* St108 were obtained from the chair of Phytopathology at the TUM (Munich, Germany) (Leiminger et al., 2016). The strain was originally collected from potato, though *A. solani* strains are known to be generalists with strains capable of infection both potato and tomato. *A. solani* and cultivated on SNA (Salt Nutrient Agar) plates (at 22 °C, 12 h UV-A light, 12 h darkness and 85% humidity) for three weeks to induce sporulation. We harvested the spores by submerging the cultures in with ddH_2_0 and scratching the mycelium off the agar with a microscope slide. The solution was filtered through four layers of mesh (grade 60, approximately 12 × 12 threads per cm^2^) and diluted to a concentration of 5,000 spores per ml. Each leaflet was infected with a 10 µl droplet.

#### Phytophthora infestans

We obtained late blight pathogen *P. infestans* strain EC1 from the James Hutton Institute (Dundee, UK). Although originally obtained from potato, samples belonging to the EC1 lineage have been found on other solaneceous hosts (Vargas et al., 2008). It was cultivated on RyeB agar, incubated six days at RT in darkness, three days at RT in daylight. We scratched the sporangia from the plate with ice cold water using a pipette tip and stored it at 4 °C until further use (up to three hours). The solution was diluted to 2,000–3,000 sporangia per ml and the leaflets were infected with 5 µl of this solution.

#### *Fusarium* sp

*Fusarium* infected lesions were identified on a few detached *S. chilense* leaves from our glasshouse. These lesions were extracted and re-cultivated for several rounds on Potato-Dextrose-Agar (PDA) for clean-up. Microscopic observations and sequence analysis of cloned Beta-Tubulin genes from several individual cultures confirmed that the cultures were axenic (Data S7). We used BLAST with the said Tubulin sequences to search against the fungidb.org database and to confirm the genus. The clean *Fusarium* was grown on PDA for a minimum of four days at RT. Spores were harvested by adding ddH_2_O and aspirating the liquid. The spores were diluted to 2 ×10^5^–5 ×10^5^ spores per ml and we infected the individual leaflets with 5 µl of this solution.

All protocols for pathogen cultivation, including ingredients for the growth media can be found in more detail at https://www.protocols.io/view/Plant-Pathogen-Cultivation-fmkbk4w.

### Leaf pre-treatment

In some wild tomato species (e.g., *S. pennellii)*, thick and sticky surface coatings have a dramatic effect on pathogen ingress. In *S. chilense,* surface coatings are notably less thick, and resemble those of cultivated tomato. However, the selected *S. chilense* populations have slightly different leaf morphology. Different amounts of wax could be present and could cause differences in infection rates between the populations. We aimed to look at differences in molecular defence rather than physical barriers preventing hyphal ingression. Hence, to minimise the effect of differences in surface coating, we washed the leaves briefly in 70% ethanol, followed by rinsing with distilled water and a brief drying period before infection. This ethanol treatment has the added effect of surface sterilisation of the leaves, preventing bacterial contamination. The effects of *S. chilense* surface sterilisation and washing off the wax is noticeable during infection, but not as dramatic as with *S pennellii* (Data S1).

### Infection assays

To assess the infection rate of the different pathogens, we performed spot inoculations on detached leaves. Each spot was scored as either negative (no symptoms or only a small hypersensitive response lesion) or positive (larger (expanding) lesions or full proliferation of the pathogen) (Fig. 1B). To minimise the effect of variation between plants within one population, we randomly collected leaves with the same age from eight to ten plants per population and shuffled them. We then drew the leaves from that mix to distribute them over up to nine boxes for each infection experiment. Each box contained 16 leaves,in four columns with four rows. Each column corresponding to a different population. Each box contained different combinations of populations (Fig. 1C). Box number and leaf position were marked to later rule out possible effects. For each pathogen 16–24 leaves—about 100 leaflets—were infected for each population. Over the course of several months, we performed four biological replicates for each population, accumulating to about 450–500 infected leaflets per population per pathogen. The *Alternaria* infections were done on the adaxial side of the leaves, *Phytophthora* and *Fusarium* infections were done on the abaxial side of the leaves. The leaves were incubated at RT and scored after six to eight days, dependent seasonal variability of temperature and light conditions in the lab, which slightly affected the rate of symptom development.

### Data analysis

All data analysis was done using R (version 3.2.3) (R foundation for statistical computing). Generalised Linear Mixed Models (GLMM) were made using the glmer option from the package lme4 (Bates et al., 2015, p. 4). To construct GLMM we used a binomial variable (y) consisting of the number of successful and unsuccessful infection events per leaf. The GLMM were constructed taking the leaf position in the box (leaf) and a combination of the box number and experimental date (exp:box) into account as random effects. For our first model populations names were used as fixed effects. (model 1 = *y* ∼ accession + (1|leaf) + (1|exp : box)). For the next models, we hierarchically tested different climatic and geographical parameters (e.g., model 2 = *y* ∼ geographic1 + climatic1 + climatic2 + (1|leaf) + (1|exp : box)). Pairwise comparisons were examined using an implementation of Tukey Honest Significant Difference test as provided by function glht from the R package multcomp (Hothorn, Bretz & Westfall, 2008). glht allows post-hoc hypothesis testing, similar to THSD, but is more suitable for general linearised (mixed) models. The boxplots were drawn using the package ggplot2 (Wickham, 2009, p. 2). All packages are available through CRAN.

### Distribution map and geographical characteristics

Geographical data for all populations were obtained from the Tomato Genome Resource Centre. Climatic data were extracted from the http://worldclim.org/ database. The species distribution map was drawn using the maps package (Becker & Wilks, 1995) in R. All geographic and climate data used can be found in Data S2.

## Results

### *S. chilense* populations show different resistant properties against different pathogens

**Figure 2:**
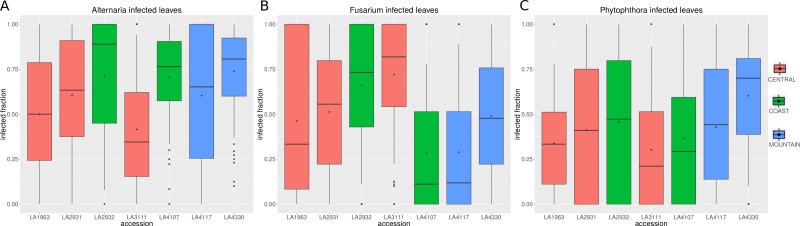
Infected leaf fraction for different *S. chilense* populations. The boxplots show the median and 1st and 3rd quartile of the infected fractions per leaf for (A) *Alternaria*, (B) *Fusarium* and (C) *Phytophthora*. The black dots represent the mean value for the infections. The *Y* axis ranges from 0 (no infected leaflets on a leaf) to 1 (all leaflets show infection). On the *X* axis, each population is represented. The colours correspond to the geographic regions as depicted in Fig. 1.

To test infection rates, we infected individual leaflets for up to 16 leaves of each *S. chilense* population with *Alternaria solani* (st108) and counted the occurrence of infected leaflets per leaf, as this represents the success rate of the pathogen to establish itself and overcome genetic resistance. We scored infection events as either negative (no infection or clear small necrotic lesions, indicating a hypersensitive response) or positive (ranging from growth just outside the droplet area up to full infection of the leaflet) (Fig. 1B) (All raw data can be found in Data S3). We observed variation within each population. In almost all instances at least one leaf was fully infected whereas another was completely resistant. These outliers have large effect on the calculated mean fraction. To allow good judgement we report the 1st and 3rd quartile, the median value as well as the mean value for each population (Fig. 2). The mean and median of the infected fractions range from 0.35 and 0.42 for LA3111 to 0.74 and 0.81 for LA4330 or 0.67 and 0.82 for LA2932.

To test the robustness of our method, we did an additional infection with a second strain of *Alternaria* (B055). The overall infection rates were lower in this set of experiments (median of 0.54 compared to 0.62), however Data S4 shows that just like for strain st108, LA3111 is the least infected population with a mean of 0.40 and LA4330 and LA2932 have a high median, with an infected fraction of 0.70 or 0.73 respectively.

With *Fusarium* we also saw differences in the infected fraction amongst populations. Interestingly LA3111 was in this case the most infected population (mean: 0.72, median: 082) whereas LA4107 was the least susceptible (mean = 0.28, median = 0.11).

Finally, for *P. infestans*, the infected fractions again showed a different pattern. There was a larger spread of the data as can be seen by the increased distance between the 1st and 3rd quartile and the lowest and highest mean and median fraction were closer together, ranging from 0.30 and 0.21 for LA3111 to 0.60 and 0.70 for LA4330 (Fig. 2C). LA3111, one population that seemed particularly resistant against *Alternaria* and *Phytophthora* seems to be the most susceptible to *Fusarium*

To test the significance of the differences and the effect of the different populations on infection, we constructed a general linearised mixed model (GLMM). We assigned experimental parameters (data, box and leaf number) as random effects and tested whether there were significant differences between the populations for each infecting species by looking at the infection counts (y) per leaf. These models showed that indeed there are highly significant differences (*p* < 0.00001) in infection rates between some populations for all three pathogens tested (Data S2).

### Pairwise comparisons reveal individual differences between different pathogens

**Figure 3:**
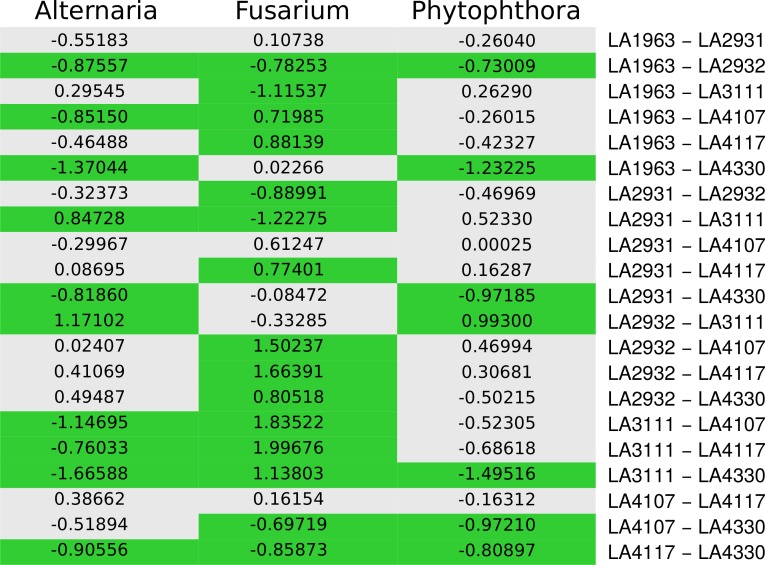
Populations with significant different infected fractions. Graph depicting whether a pairwise difference shows a significant result for *Alternaria solani*, *Fusarium sp.* and *Phytophthora infestans*. Each row represents a pairwise comparison. Green cells represent a significant difference (*p* < 0.001 after multiple testing correction) and the numbers represent the estimated effect, with negative numbers indicating that the population mentioned on the left is less resistant than the one on the right.

To further determine which populations were different from each other, we performed pairwise comparisons using a variant of Tukey’s Honest Significant Difference test (see methods). The observed pairwise differences were clearly distinct between the three pathogens. Figure 3 shows a summary of the pairwise differences, with corresponding difference estimates for each comparison. Cells with significant differences (*p* < 0.001) are highlighted in green. All pairwise differences with their 95% confidence intervals are plotted in Data S5. Of the 63 pairwise comparisons, 32 showed a significant difference in infection ratio. Overall, there were more significant differences between populations when it came to *Fusarium* infection (15) than to Alternaria infection (10) or Phytophthora (7). Interestingly, some populations showed the same result for all pathogens: there are no differences between LA1963 and LA2931 (both central) nor for LA2931 and LA4107 (central and south coast) or LA4107 and LA4117 (south coast and south mountain). Also, LA1963 was always more susceptible than LA2932, and LA4117 was always more susceptible than LA4330. In some cases a population in a pair was more resistant to one pathogen and more susceptible to another. LA4330 was more resistant than LA3111 to *Fusarium*, but less resistant to *Alternaria* and *Phytophthora*.

### A mix of climatic and geographic variables affect pathogen resistance

To see whether a change in certain geographic and climatic conditions could be linked to an increase or decrease of resistance rates between populations, we built new GLMM using such data. First we made a simple model for resistance to *Alternaria*, testing the infection counts (y) against either latitude or longitude, a combination of both or an interaction of both. This showed that both latitude and longitude had a significant effect (*p* < 0.001, Table 1). The quality of the models is reported by the statistical software using the Akaike Information Criterion (AIC), where a lower AIC, indicates a better relative quality of the model. A model with both parameters showed a better AIC. However, a model with an interaction only shows significance of the latitude parameter. We extended the model to include both parameters (longitude + latitude) as well as several environmental parameters. We obtained the best AIC (2641.8) for a model containing altitude, annual precipitation, the temperature in the wettest and the temperature in the coldest quarter. Additions of other climatic data did not yield an improvement of the model. A selection of tested models with their AIC and significance is shown in Table 1. When closely investigating the best fitting model for resistance to *Alternaria* (Table 1, model 6), we noted that whereas all variables contribute significantly to the model, the estimated effect size differs greatly. Table 2 shows that of all effects, longitude was the strongest effect, followed by the mean minimum temperature in winter (TempB), the annual precipitation and altitude. It should be noted that models that only take temperature effects into account did not account for significance (Table 1). A GLMM with the infection counts set against the previously identified genetic groups (y ∼group), yielded a higher and thus worse AIC (2705). The model with the populations yields an as good AIC as the one with six climatic variables. This suggested that no single variable has a strong, exclusive correlation to infection rate and that each population represented its own micro environment with specific geographic and climate parameters that are all of influence.

**Table 1:** Summary of GLMM results. AIC is a measure of relative quality of the model, with a lower AIC (within one species) indicating a better model. AICs in bold represent models that are significant (*p* < 0.001). TempA denotes the temperature in the wettest quarter, TempB in the coldest quarter and TempC the annual mean temperature.

	Model	*Alternaria*	*Fusarium*	*Phytophthora*
1	y ∼accession	**2641.8**	**2307.6**	**1893.3**
2	y ∼Lat	**2708.6**	**2431.3**	**1958.3**
3	y ∼Long	**2815.1**	**2490.8**	**1965.8**
4	y ∼Long + Lat	**2703.9**	**2420.6**	**1945.4**
5	y ∼Long*Lat	2705.8	2419.1	1947.4
6	y ∼Long + Lat + Alt + AnnPrecip + TempA + TempB	**2641.8**	**2307.6**	**1893.3**
6b	y ∼Long + AnnPrecip + TempA + TempB	2753.2	**2303.6**	**1927.7**
7	y ∼Altitude	2843.3	2503.5	1985.1
8	y ∼TempA	2842.2	2506.5	1987.0
9	y ∼TempB	2842.2	2506.5	1981.3
10	y ∼TempC	2842.2	2506.5	1984.1
11	y ∼AnnPreci	**2784.2**	**2457.1**	**1968.4**
12	y ∼group	**2705.0**	**2489.7**	**1930.2**

**Table 2:** Effects and significance of environmental variables, estimated size of the effect and the significance for all variables used in model 6 (Table 1). *P*-values in bold are deemed significant. TempA denotes the temperature in the wettest quarter, TempB in the coldest quarter.

Variable	*Alternaria*		*Fusarium*		*Phytophthora*	
	Effect estimate	*P*-value	Effect estimate	*P*-value	Effect estimate	*P*-value
Latitude	−0.5	<**0.001**	−0.1	0.8	−0.3	<**0.001**
Longitude	17.4	<**0.001**	7.9	**0.007**	17.3	<**0.001**
Altitude	−7.4	<**0.001**	0.0	0.99	−6.5	<**0.001**
Precipitation	9.1	<**0.001**	3.8	**0.02**	8.3	<**0.001**
TempA	3.2	<**0.001**	1.5	**0.004**	3.1	<**0.001**
TempB	10.2	<**0.001**	7.8	<**0.001**	10.6	<**0.001**

Similar to resistance to *Alternaria*, we tested all variables for resistance to *Phytophthora* and resistance to *Fusarium*. The pattern seen for resistance to *Phytophthora* is almost identical to that of * Alternaria* resistance. The AIC values are generally lower, but the trends are the same. Interestingly, *Fusarium* resistance showed a different picture. Whereas longitude was still the strongest effect, its effect and significance were lower and the temperature in the coldest quarter of the year had a relatively larger effect. The effect of altitude was not significant and differences in annual precipitation had a nearly negligible effect as well. In fact, a simplified model (Table 1, model 6b) that excludes the insignificant parameters had a better fit. As with resistance to *Alternaria*, the model testing for the genetic group effect showed a lesser fit than the model per population (results for selected models can be found in Data S2).

## Discussion

The wild tomato *Solanum chilense* grows in a variety of habitats in Chile and Peru, ranging from lower coastal areas to very high altitudes (>3,000 m). These populations experience considerable variation in geographic parameters like precipitation and temperatures. It is known that *S. chilense* has a clear demographic pattern and signs of adaptations to climatic differences between different populations (Fischer et al., 2011; Fischer et al., 2013; Nosenko et al., 2016). A demographic pattern of North–South colonisation is observable with larger and more diverse populations in the north of the range and smaller and less diverse populations in the south. In addition, there is little to no genetic exchange between some of the southern most populations that are separated by the extremely dry Atacama desert. This leads to the conclusion that *S. chilense* can be divided in a northern, a central and two southern genotype groups (Böndel et al., 2015).

We hypothesised that pathogen pressures must differ a lot between such diverse geographical locations and as such *S. chilense* should show variation in resistance to common plant pathogens between the different populations. The underlying assumption is that at the evolutionary time scale of thousands of years, climatic conditions generate different selective pressures and presence/absence of pathogens, which then drives the evolution of the plant immune genes along different evolutionary paths in different regions. This heterogeneity in space may be seen as variation in functionality or duplication of resistance genes (so-called R-genes) and different levels of quantitative resistance across populations depending on pathogen availability and trade-offs for costs of resistance (Moreno-Gamez, Stephan & Tellier, 2013). The result of this evolutionary process was tested by performing infection assays in a common environment to assess the degree of resistance to three *Solanum* pathogens. We observed clear differences between the infection success rates of the three pathogens on the different *S. chilense* populations, indeed suggesting local adaptations to pathogen pressure. We could only observe a clear separation between three regional *S. chilense* genotype groups for resistance to *Alternaria*, where the central populations are more susceptible than those from groups in the south. This shows that the genotype groups can be seen as an indicator of resistance against *Alternaria*. For resistance to the other pathogens large within-group differences exist and the genotype groups cannot be considered an indicator of resistance. Pairwise comparisons confirmed that outcomes differ within groups and between pathogens. For example, a pair that shows significant differences for resistance to *Phytophthora* and * Alternaria* (LA1963–LA4330) does not show differences in infection rate for *Fusarium.* Very strong pairwise differences can even be seen within the previously identified genotype groups (e.g., LA2932–LA4107 with *Fusarium*). We also showed that there are no populations that are in general more resistant (e.g., against all pathogens). For example, LA3111 is particularly resistant against *Fusarium*, but the most susceptible to *Phytophthora* and *Alternaria*.

We used a GLMM to test which factors might contribute to these differences. Interestingly, whereas the species *S. chilense* as a whole, shows a strong north–south demography, our analyses show that in models with multiple variables not latitude, but longitude has a very strong effect on infection rates. This could on the one hand be explained by the absence of the northern-most group in our analysis. On the other hand, a more likely explanation is the bigger geographic and associated climatic difference in the east–west gradient of the species, with low altitude coastal areas in the west, and high mountains in the east. Temperature differences can have large effects on the prevalence of pathogen populations as shown for wild plant-pathosystems (Laine, 2008) and also on crops, pathogens show adaptation to different climates (Mboup et al., 2012; Stefansson, McDonald & Willi, 2013). The mountainous areas in our study have particularly cold winters and relatively low mean temperatures in summer, which could be detrimental for pathogen survival or slow its growth and thus reduce pathogen pressure, which in turn might lead to loss of resistance in the plants.

Our results show indeed that inclusion of climatic variables for “temperature in winter” as well as “temperature in the wettest quarter” greatly improve our models and have a significant effect on infection rate for both *A. solani* and *P. infestans*. The importance of overwintering inoculum has previously been shown to be a main predictor for *Podosphaera plantaginis* epidemics on *Plantago lanceolata* in the next growing season (Soubeyrand et al., 2009). However, it must be noted that models that only incorporate winter temperature or indeed any other single climatic variables effects did not show any significance. This is in line with a between species comparison for wild potato (Spooner et al., 2009) and might be related to the fact that some higher altitude locations also have the highest annual precipitation rates. For example for *P. infestans* a relative high humidity has large positive effects on successful sporulation (Harrison & Lowe, 1989), which could counter the negative temperature effects.

Our climate data were extracted from http://worldclim.org/ and might not provide the whole picture. For example, precipitation data might be accurate, but do not take into account a common sea-fog phenomenon that can be observed along the coast of Chile and Peru (Cereceda & Schemenauer, 1991; Schemenauer & Cereceda, 1992). This fog increases the local humidity for several hours up to several days in certain “fog basins”. Similarly, no data is available on any nearby streams, rivers or irrigation canals for any of the populations. For some populations, a note is available for the state of the site at the time of collection (e.g., “dry quebrada”), but it remains unknown whether these features are constant or changed shortly before collection.

The best fitted models incorporate five climatic and geographic variables. Adding more variables did not improve the model, mainly due to the correlations between the available climate data. The strongest effects were observed for combinations of longitude and latitude together with climatic variables, indicating that one or two variables alone do not determine pathogen resistance. The latitude effect, which can be observed in the evolution of the species as a whole, seems to be less strong in our analyses, where longitude plays a larger role. Overall, our results indicate that indeed *S. chilense* populations show variations in pathogen resistance, which are possibly the result of adaptations to local pathogen pressures. The mosaic-like structure of our results indicates that these resistances are likely caused by a multitude of factors. These findings are in line with several inter-species studies in wild potato, where no correlation could be found between geographical location of the species and resistance against *P. infestans* (Khiutti et al., 2015) or *A. solani* (Jansky, Simon & Spooner, 2008). To further unravel the combination of factors contributing to local variations, new sampling excursions would be required, that not just collect plant and pathogen, but also measure local geographic and climatic parameters.

Several mechanisms have been theoretically proposed to generate stable long term polymorphism at host resistance and pathogen infectivity loci. Notably, many of those are linked to the environmental heterogeneity across populations. Here we show that (1) multiple environmental factors are required to improve the GLMM (2) infection rates differ between populations and even within previously genetic groups and (3) *S. chilense* populations show individual patterns in response to the three different pathogens. Therefore we conclude on (1) the existence of possible GxGxE interactions for given host-pathogen interactions, (2) heterogeneity in disease incidence and prevalence across habitats, and most interestingly (3) a likely geographic mosaic of exposure to different pathogens. However, additional experiments with locally collected pathogens are required to confirm these conclusions.

The presence-absence of different above- and below-ground pathogens on the same plants may be a key component of wild systems generating scenarios such as co-infection (Susi et al., 2015), cross-immunity or facilitation (Tack et al., 2015), with consequences for the genomics of pathogens (McMullan et al., 2015). Our research did not yet focus on any genetic differences underlying the variation in infection rates and linking phenotype to genotype should be one of the follow-up projects. Identification of the genes involved in these resistance variations could also help to identify which plant defence mechanisms are affected between populations and if there are indeed evolutionary differences between defence pathways in non-host resistance compared to resistance variation within or in closely related species (Schulze-Lefert & Panstruga, 2011; Stam et al., 2014). We have recently shown that targeted resequencing of genes of interest (e.g., R-genes) can be a potent tool to calculate evolutionary parameters of gene families of interest in wild tomato (Stam, Scheikl & Tellier, 2016). Such resequencing studies could thus help to pinpoint how molecular mechanisms are affected by different pathogens as well as climatic variables.

## Conclusions

Differences in pathogen disease resistance have been well described between many wild crop relatives. Here we presented a phenotypic study that shows large variation in resistance against three common tomato pathogens between populations of the wild tomato species *S. chilense*. We showed that there are clear differences between individual populations. Using generalised linear mixed models, we show that this variation does not follow a simple geographical cline, that multiple climatic factors are needed to explain parts of the variation and that even within previously identified genotype groups resistance properties can differ dramatically. Our study confirms a mosaic pattern in resistance properties within one species and can form the starting point for studies unravelling environmental effects on said properties as well as the genetic and molecular mechanisms involved in plant-pathogen coevolution.

##  Supplemental Information

10.7717/peerj.2910/supp-1 Data S1 Surface Sterilisation Sterilisation of leaf surface area has moderate effects on infection of *S. chilense.* (A) *S. pennellii* leaf, left washed with distilled water, right washed with 70% EtOH. (B) Water (left), EtOH (right) washed of *S. chilense* LA4330 leaves.

10.7717/peerj.2910/supp-2 Data S2 Climatic Data

10.7717/peerj.2910/supp-3 Data S3 Raw Infection Data: infection scoring for each pathogen

10.7717/peerj.2910/supp-4 Data S4 Alternaria B055 infection Infected fraction of seven *S. chilense* populations for Alternaria solani strain B055. The boxplots show the median and 1st and 3rd quartile of the infected fractions per leaf. The *Y* axis ranges from 0 (no infected leaflets on a leaf) to 1 (all leaflets show infection). On the *X* axis, each population is represented. The colours correspond to the geographic regions as depicted in Fig. 1.

10.7717/peerj.2910/supp-5 Data S5 Pairwise comparisons for all infection experiments Results of Tukey’s Honest Significant Differences test for pairwise comparisons between all populations for (A) Alternaria (B) Fusarium (C) Phytophthora. The *Y*-axis indicates the individual comparisons, the *X*-axis shows the observed difference (vertical lines), with a 95% confidence interval (whiskers). Differences are considered significant if the whiskers do not cross the line at 0.

10.7717/peerj.2910/supp-6 Data S6 Summaries for Generalised Mixed Linear Models used in this study

10.7717/peerj.2910/supp-7 Data S7 Fusarium spores and (partial) sequences for cloned B-Tubulin as used to confirm the genus of the fungus

## Additional Information and Declarations

### Data Availability

The following information was supplied regarding data availability: The raw data has been supplied as a Supplementary File.
