# Supplementary figures and images for: The wild tomato species Solanum chilense shows variation in pathogen resistance between geographically distinct populations
Source: PeerJ. 2017 Jan 18;5:e2910. doi: 10.7717/peerj.2910 (PMC5248578; doi:10.7717/peerj.2910)

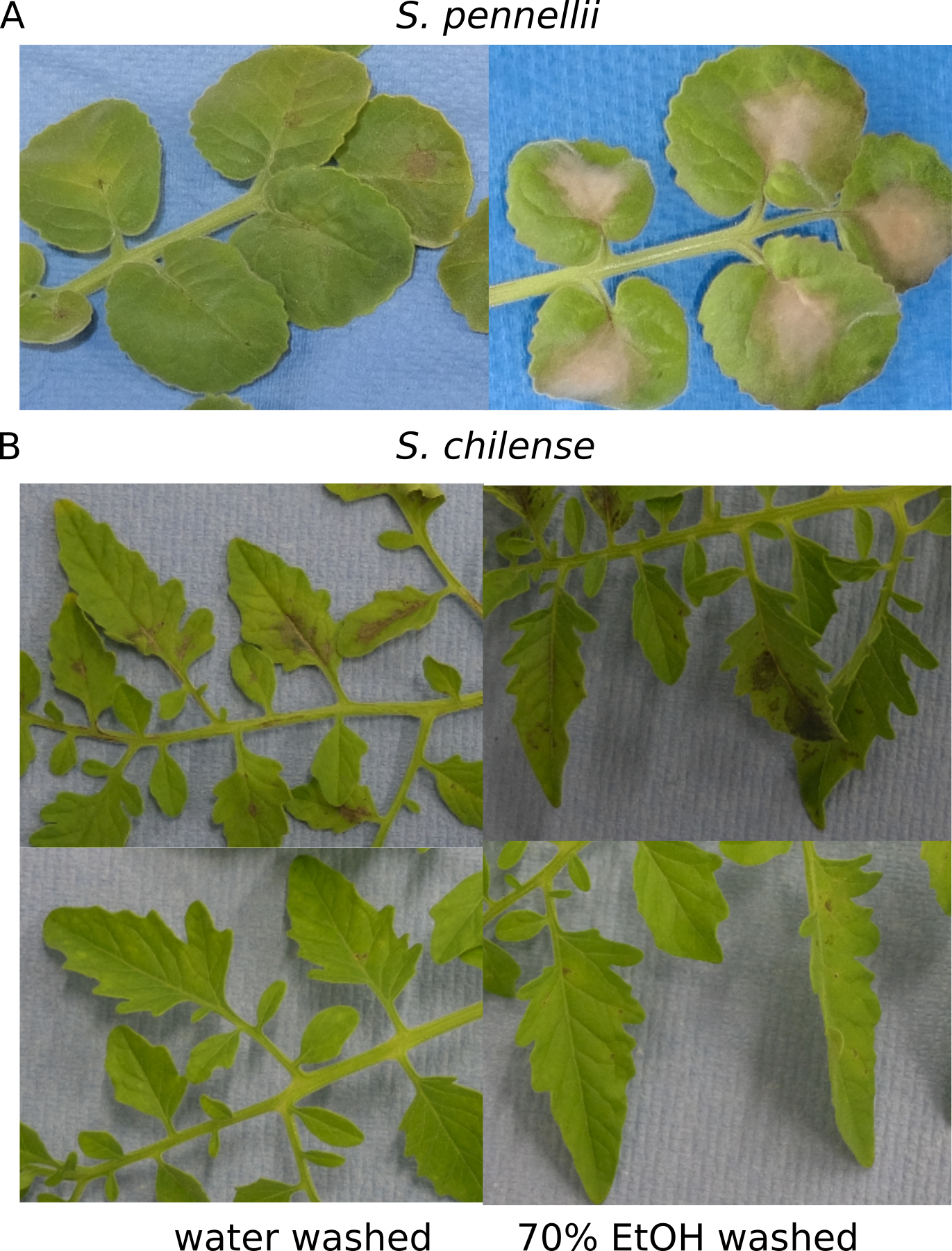

Supplement: Data S1 — Sterilisation of leaf surface area has moderate effects on infection of S. chilense. (A) S. pennellii leaf, left washed with distilled water, right washed with 70% EtOH. (B) Water (left), EtOH (right) washed of S. chilense LA4330 leaves. [file peerj-05-2910-s001.png]

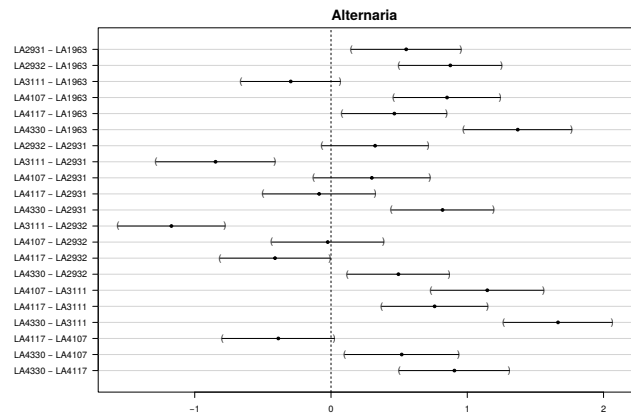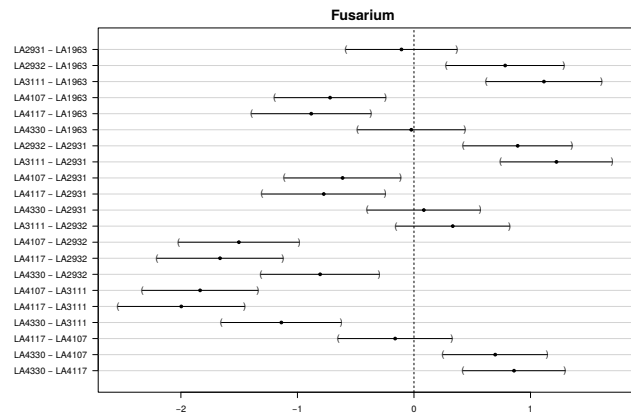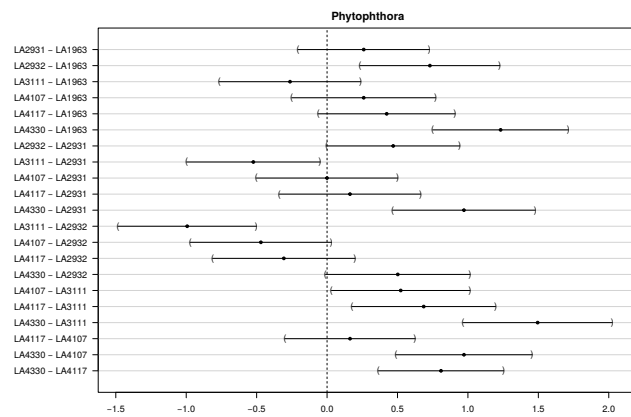

Supplement: Data S5 — Results of Tukey’s Honest Significant Differences test for pairwise comparisons between all populations for (A) Alternaria (B) Fusarium (C) Phytophthora. The Y-axis indicates the individual comparisons, the X-axis shows the observed difference (vertical lines), with a 95% confidence interval (whiskers). Differences are considered significant if the whiskers do not cross the line at 0. [file peerj-05-2910-s005.pdf]

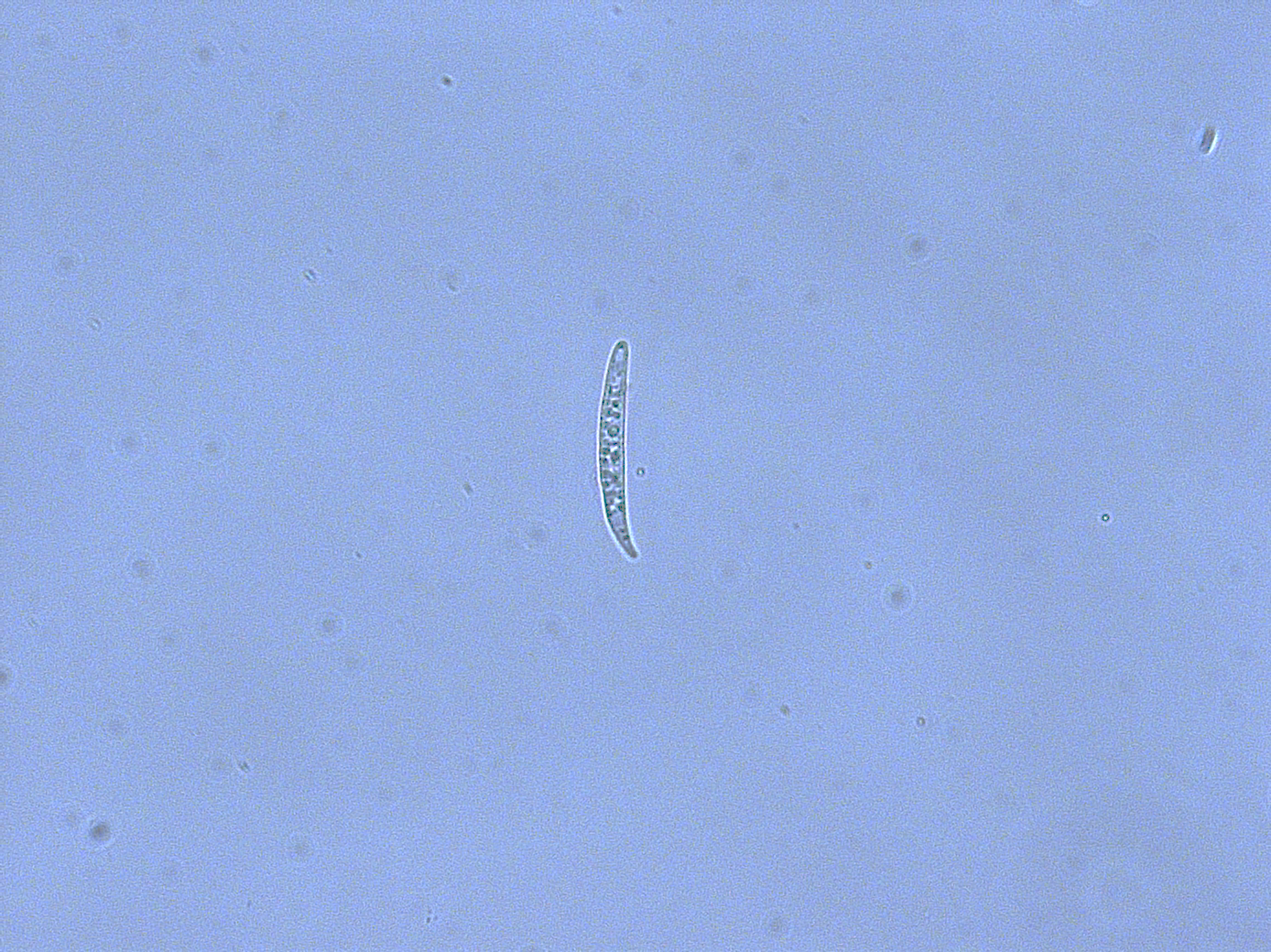

Supplement: Data S7 [file peerj-05-2910-s007.gz › SNAP-111751-0035.tif]

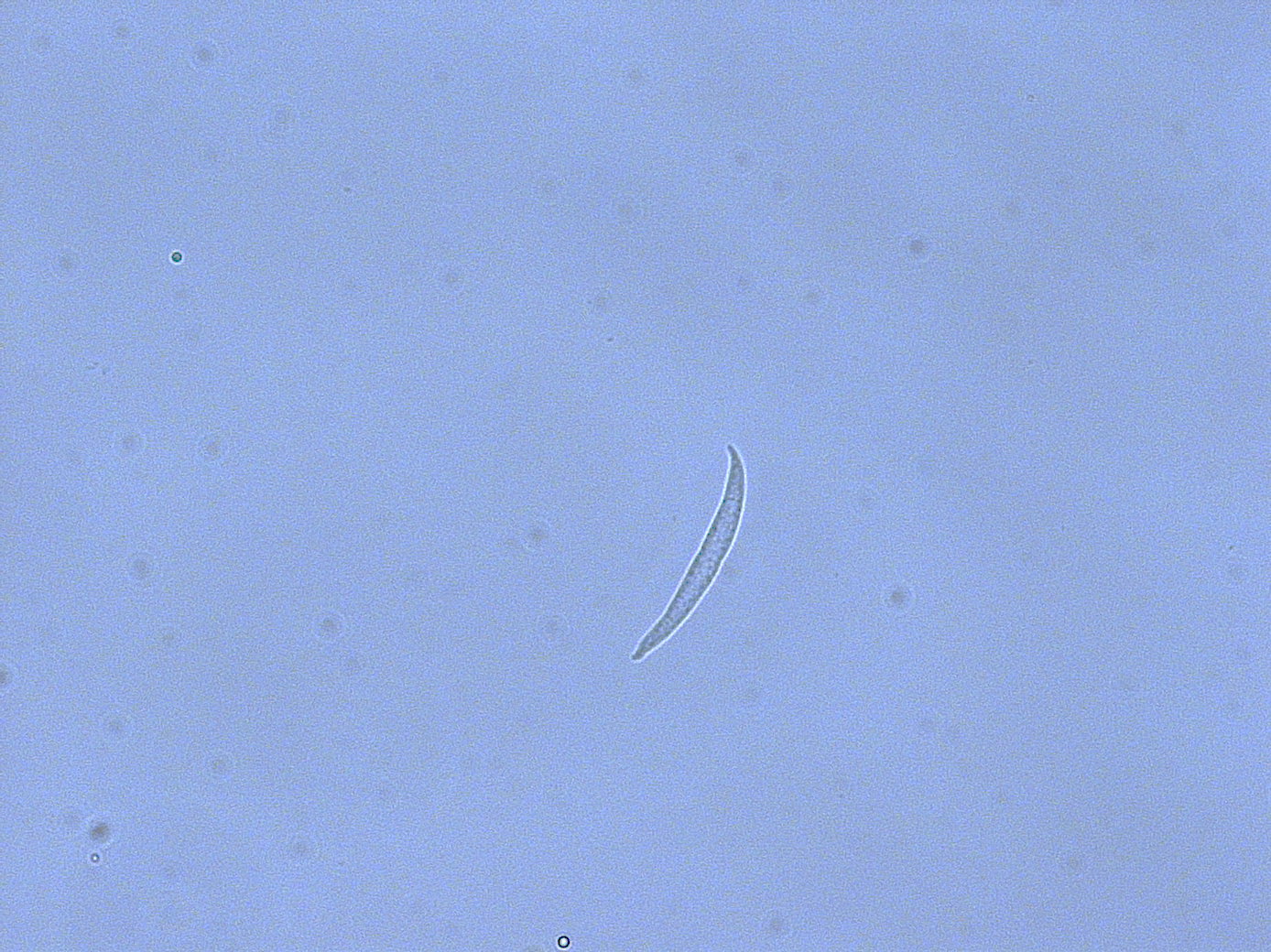

Supplement: Data S7 [file peerj-05-2910-s007.gz › SNAP-112630-0036.tif]

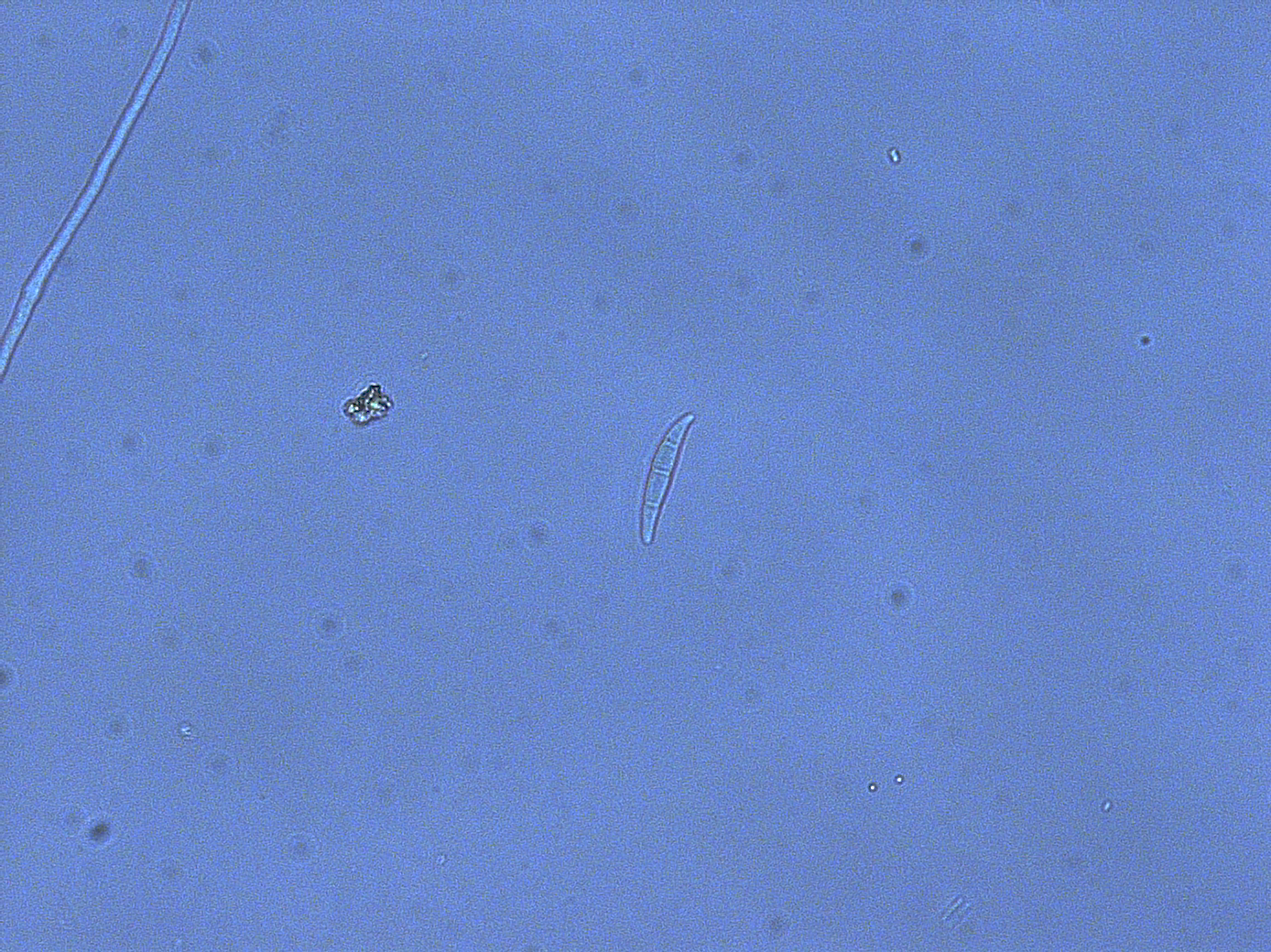

Supplement: Data S7 [file peerj-05-2910-s007.gz › SNAP-110258-0021.tif]
