# Supplementary material for: The wild tomato species Solanum chilense shows variation in pathogen resistance between geographically distinct populations
Source: PeerJ. 2017 Jan 18;5:e2910. doi: 10.7717/peerj.2910 (PMC5248578; doi:10.7717/peerj.2910)
Supplement: Data S4 — Infected fraction of seven S. chilense populations for Alternaria solani strain B055. The boxplots show the median and 1st and 3rd quartile of the infected fractions per leaf. The Y axis ranges from 0 (no infected leaflets on a leaf) to 1 (all leaflets show infection). On the X axis, each population is represented. The colours correspond to the geographic regions as depicted in Fig. 1. [file peerj-05-2910-s004.pdf]

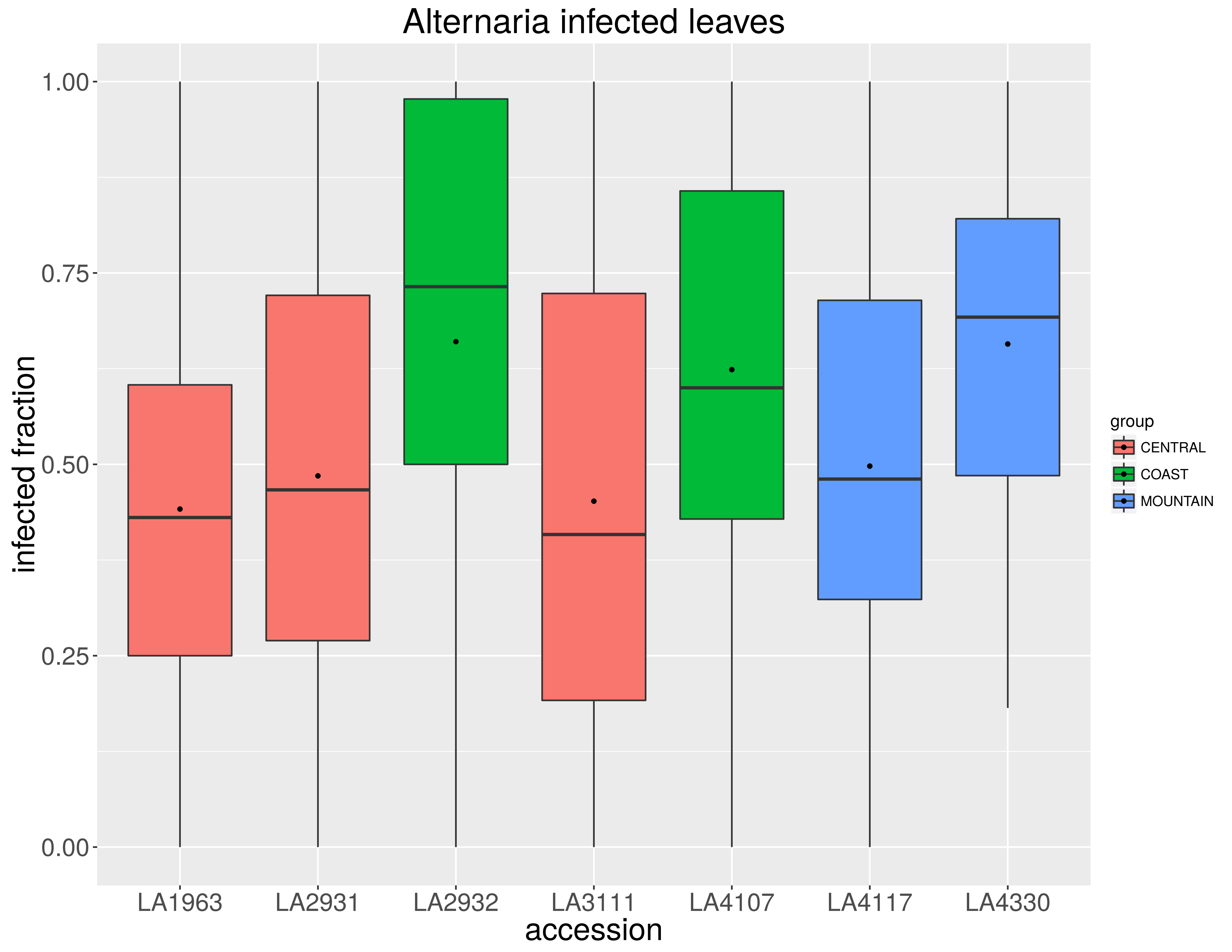
Alternaria infected leaves
infected fraction
1.00
0.75
0.50
0.25
0.00
LA1963
LA2931
LA2932
LA3111
LA4107
LA4117
LA4330
accession
group
CENTRAL
COAST
MOUNTAIN
